# Metabolic Dysfunction-Associated Steatotic Liver Disease in a Patient with Phelan–McDermid Syndrome

**DOI:** 10.3390/life15101586

**Published:** 2025-10-11

**Authors:** Luigi Boccuto, Giuseppe Guido Maria Scarlata, Bridgette A. Moffitt, Sara M. Sarasua, Katy Phelan, Curtis Rogers, Ludovico Abenavoli

**Affiliations:** 1 Healthcare Genetics and Genomics Program, School of Nursing, College of Behavioral, Social and Health Sciences, Clemson University, Clemson, SC 29634, USA; lboccut@clemson.edu (L.B.); smsaras@clemson.edu (S.M.S.); 2 Department of Health Sciences, University “Magna Græcia”, 88100 Catanzaro, Italy; giuseppeguidomaria.scarlata@unicz.it; 3 Genetics Laboratory, Florida Cancer Specialists and Research Institute, Fort Myers, FL 33908, USA; kphelan@flcancer.com; 4 Greenwood Genetic Center, Greenville, SC 29605, USA; rcrgenetics@gmail.com

**Keywords:** guidelines, diagnosis, treatment, liver steatosis, genetic mutations, pathogenesis

## Abstract

Background: Phelan–McDermid syndrome (PMS), caused by *SHANK3* variants or 22q13.3 deletions, often includes systemic features such as gastrointestinal and hepatic abnormalities. This study highlights the overlap between PMS and metabolic-associated steatotic liver disease (MASLD), focusing on *PNPLA3* variants and underscoring the need for structured metabolic monitoring. Methods: We describe a 25-year-old male with PMS due to a 22q13.33 microdeletion involving *SHANK3*. He exhibited developmental delay, seizures, and hypotonia. Genetic testing revealed homozygosity for the *PNPLA3* p.I148M variant. Clinical, anthropometric, biochemical, imaging, and metabolic investigations were performed, including liver ultrasound and metabolic profiling of lymphoblastoid cell lines. Results: Ultrasound revealed moderate hepatic steatosis consistent with MASLD. After ursodeoxycholic acid treatment and a Mediterranean-style diet, steatosis improved to mild. Metabolic profiling demonstrated increased nicotinamide adenine dinucleotide generation under metabolic stimuli, suggesting altered energy homeostasis. Conclusions: We highlight the contribution of *PNPLA3* to MASLD in PMS and support systematic hepatic monitoring. Genotype–phenotype associations in PMS may provide insights relevant to MASLD research and clinical management.

## 1. Introduction

Phelan–McDermid syndrome (PMS), also known as 22q13 deletion syndrome, is a rare genetic disorder caused by deletions in chromosome 22q13.3 or mutations in the *SHANK3* gene located in the same region [1]. This syndrome is characterized by a wide spectrum of clinical manifestations (Table 1), including intellectual disability, delayed or absent speech, and various physical abnormalities. PMS is considered a significant medical condition due to its profound impact on the quality of life of affected individuals and their families [2]. Epidemiologically, PMS is estimated to occur in 1 in 8000 to 1 in 15,000 individuals, making it a relatively uncommon but noteworthy genetic disorder that necessitates further research and awareness [3]. The pathogenesis of PMS is primarily linked to the disruption of the *SHANK3* gene, which plays a crucial role in the development and function of synapses in the brain. This disruption leads to the neurological and developmental issues observed in PMS patients. The clinical presentation of PMS varies widely but often includes neurological traits such as developmental delay, language delay, motor impairment, hypotonia, seizures, sleep disturbances, and autism spectrum disorder [1,2,3,4]. Systemic features are also commonly reported and include gastrointestinal issues, renal disorders and lymphedema [1,5,6,7,8]. Currently, there is no cure for PMS, and treatment is primarily supportive, focusing on managing symptoms and improving the quality of life [7,9]. Interventions may include physical therapy, speech therapy, occupational therapy, and medications to control seizures and other associated conditions [9,10]. Among the non-neurological features of PMS, gastrointestinal issues are often overlooked and underdiagnosed due to the language impairment of the affected individuals and the difficulties in following a regular dietary regimen in the presence of complicating factors such as hypotonia and behavioral issues. Moreover, while digestive problems associated with gastroesophageal reflux or abnormal intestinal motility may be managed by parents or caregivers, signs and symptoms of liver disease often go unnoticed unless tests are performed, even though at least two major candidate genes for liver disease have been identified in the 22q13 region (*patatin-like phospholipase domain containing 3*, *PNPLA3*, and *cytochrome P450 2D6*, *CYP2D6*) and liver dysfunction might exert secondary effects on other PMS phenotypes [5,6,8,11]. A growing body of evidence supports the multisystemic impact of liver disease, with disruptive consequences on the cardiovascular system and lipid metabolism. Metabolic-associated steatotic liver disease (MASLD), previously known as non-alcoholic fatty liver disease (NAFLD), represents a spectrum of liver conditions ranging from simple steatosis to metabolic dysfunction-associated steatohepatitis (MASH), which can progress to fibrosis, cirrhosis, and hepatocellular carcinoma (HCC) [5]. The change in nomenclature from NAFLD to MASLD reflects a broader understanding of the disease’s metabolic roots and its association with metabolic syndrome components, such as overweight or obesity, hypertension, type 2 diabetes mellitus (T2DM), and dyslipidemia [12]. MASLD is now recognized as the most common chronic liver disease worldwide, affecting approximately 25% of the global population [13,14,15]. Emerging research highlights a potential interplay between MASLD and PMS, particularly involving the *PNPLA3* gene and its product, adiponutrin, which is known to influence lipid metabolism and liver fat accumulation. *PNPLA3* is located on chromosome 22q13.31 and is sometimes co-deleted in PMS due to deletions of 22q13. In addition to the loss of a copy of *PNPLA3*, variants can be present in the gene. Variants in the *PNPLA3* gene, especially the I148M polymorphism, have been implicated in increased susceptibility to liver disease and MASLD in particular [5,16,17,18,19]. Moreover, the *PNPLA3* I148M variant is the primary driver of weight gain following hepatitis C virus cure [20]. At the same time, a recent observational study of 298 MASLD patients underscored the impact of genetic risk on disease progression, demonstrating that the *PNPLA3* variant is linked to elevated aspartate aminotransferase levels [21]. Given that individuals with PMS may have altered metabolic profiles and a higher prevalence of obesity and related metabolic conditions, understanding the interaction between PMS and MASLD at the genetic level is crucial for developing targeted therapeutic strategies [22,23,24]. There is an urgent need to apply the new MASLD definition in patients with PMS to enhance the understanding and management of liver-related complications in this population. At the same time, there is an international consensus regarding the management of patients with PMS and NAFLD, but not for MASLD [9]. Recognizing MASLD in the context of PMS is pivotal for early diagnosis, appropriate management, and the prevention of advanced liver disease. In consideration of the pivotal role of *PNPLA3* variants in the pathogenesis of MASLD, PMS may indeed be considered a model disease that will allow researchers and physicians to explore dysmetabolic dynamics and identify molecular targets for potential treatment development. This study aims to contribute to this growing body of knowledge by presenting a case of a 25-year-old male with PMS and MASLD, highlighting the clinical, genetic, and metabolic intersections of these two conditions.

## 2. Patients and Methods

### 2.1. Clinical Presentation

**Figure 1:**
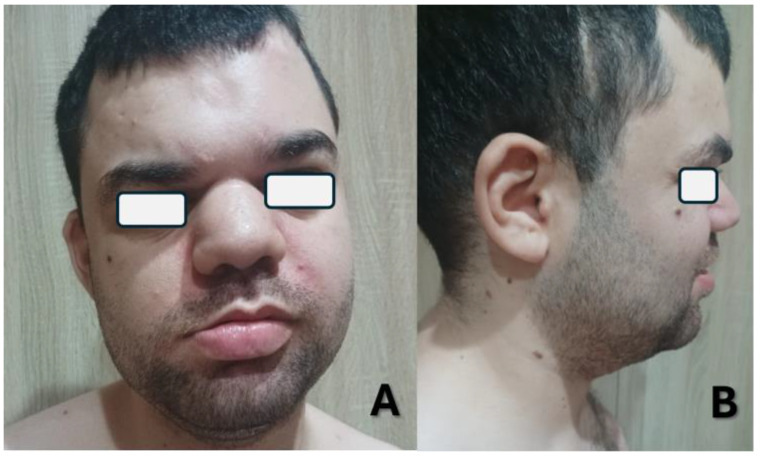
Frontal (**A**) and lateral (**B**) pictures of the index case at age 25 showing minor dysmorphic traits.

At the latest evaluation, the patient, a Caucasian 25-year-old male, presented with developmental delay, minor hypotonia, seizures, and minor facial dysmorphic traits, including facial asymmetry, bulbous nose, broad nasal root, large fleshy ears, cupid-bow sign in the upper lip, and large lower lip (Figure 1). When compared to the previous clinical report, when the subject was 20 years old, his cognitive and behavioral condition improved, and he developed a better sleep regimen [5]. However, more severe and frequent episodes of seizures—up to 2–3 events per day—have been noted until a month before the evaluation, when they seem to have responded positively to the introduction of cenobamate. Regression has been reported, mainly affecting motor and communication skills. For this reason, a sedentary lifestyle was highlighted. A negative response to gastroprotective drugs (i.e., pantoprazole) has been noted, with main consequences on behavior (agitation, irritability), hypotonia, and sleep (insomnia); the symptoms resolved drastically after the suspension of the drugs.

### 2.2. Genetic Tests

**Figure 2:**
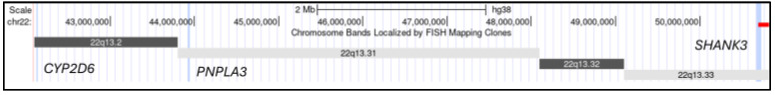
Graphic representation of the location of the main genes of interest (*CYP2D6*, *PNPLA3*, and *SHANK3*) on chromosome 22q13. The gene loci are highlighted in light blue, the deletion detected in the subject is indicated by the red bar (generated using UCSC Genome Browser, GRCh38/hg38, https://genome.ucsc.edu/index.html) (accessed on 12 May 2025).

As previously reported, genetic tests identified an approximately 100-Kb 22q13.33 deletion (chr22:51,123,491-51,224,252/hg19; chr22:51,199,330-51,299,321/hg38, Figure 2) within the *SHANK3* gene and the homozygous c. 444C > G variant (p.I148M, SNP rs738409, CADD score 13.7) in the *PNPLA3* gene [5]. Although this variant is relatively common in the general population (G: 23.25%, gnomAD v. 4.1.0), it has been thoroughly associated with various degrees of liver disease due to its predisposing effect to lipid accumulation in hepatocytes [17].

### 2.3. Lymphoblastoid Cell Lines

Lymphoblastoid cell lines (LCLs) were established from peripheral blood samples collected by venipuncture from 43 individuals, using lymphocyte immortalization Via Epstein–Barr virus for the purpose of obtaining a metabolic profile. LCLs were harvested in Sigma RPMI-1640 with 15% fetal bovine serum from Atlanta Biological (Flowery Branch, GA, USA), 2 mM L-Glutamine, 100 U/mL Penicillin, and 100 µg/mL Streptomycin from Sigma-Aldrich (St. Louis, MO, USA).

### 2.4. Metabolic Profiling via Biolog Phenotype Mammalian Microarrays

We assessed the metabolic signature of the proband’s LCLs using the Phenotype Mammalian MicroArray (PM-M) technology developed by Biolog (Hayward, CA, USA). The PM-M plates are designed to assess metabolic activity by measuring the cellular production of nicotinamide adenine dinucleotide (NADH) in the presence of different compounds. The methodology employed microplates with different molecules, which acted either as energy sources (plates PM–M1 to M4) or as metabolic effectors (plates PM–M5 to M8). Each well contained a single chemical, and the production of NADH per well was monitored using a colorimetric redox dye chemistry. In this way, NADH production was used as an indicator of metabolic dysregulation. The energy sources included carbohydrates, nucleotides, carboxylic acids, and ketone bodies in plate PM–M1, and amino acids, both alone and as dipeptides in plates PM–M2 to M4. The metabolic effectors included ions (PM–M5), hormones, growth factors, and cytokines (PM–M6 to M8). The relative absorbance (A_590_–_750_) was calculated per well, and these absorbance endpoint readings were used as a measure of metabolite usage. Readings were normalized using triplicate absorbance readings from the corresponding empty plate (plates run with just media and dye, without cells). Data were analyzed using a custom R package (version 4.1.2) and the opm R package (version 4.1.2). These values were then transformed to a logarithmic scale for analysis and compared with the average values generated by the 50 LCLs from typically developing controls. The main goal was to identify significant abnormalities in metabolic pathways and responses to effectors, specifically compounds differentially metabolized by participants and controls.

### 2.5. Statistical Analysis

Comparisons of means were performed using two-sided *t*-tests. Chi-square tests to measure associations between categorical values used the Mid-P exact two-tailed test as provided by OpenEpi [25]. The metabolic profile data were analyzed by the non-parametric Mann–Whitney two-sided test, with a cut-off of *p*-value ≤ 0.05 after applying the Benjamini–Hochberg correction (R method: p.adjust) for multiple testing (FDR).

## 3. Results

**Figure 3:**
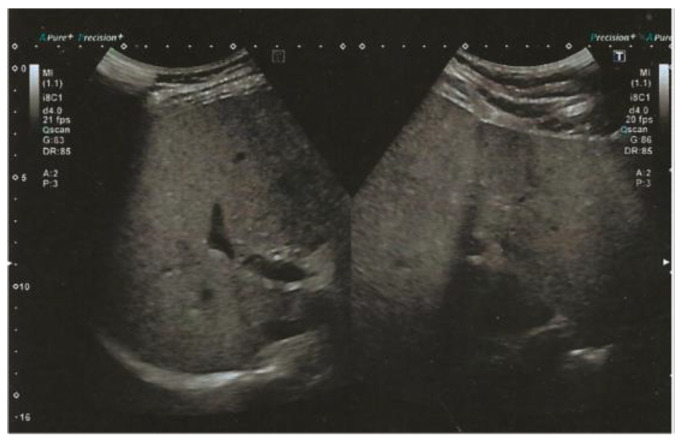
Abdominal ultrasound performed at baseline showed a diffuse increase in liver echogenicity and slight inhomogeneity, with a liver size of 16 cm, suggesting the presence of moderate liver steatosis. No additional focal liver lesions or signs of advanced liver disease are identified.

The subject is currently following a clinical follow-up protocol for his gastrointestinal symptoms, including dyspepsia and constipation. As part of such protocol and in consideration of the evidence reported regarding the *PNPLA3* p.I148M variant, the subject underwent an abdominal ultrasound, which revealed the presence of moderate liver steatosis, as shown in Figure 3, indicating a slight improvement from the previous report, most likely due to the therapeutic dietary regimen.

Due to this finding, certain anthropometric and laboratory indices associated with the diagnosis of MASLD were evaluated according to international guidelines. Specifically, steatotic liver disease (SLD) with at least one of five cardiometabolic risk factors such as (i) body mass index (BMI) ≥ 25 kg/m^2^ or waist circumference > 94 cm; (ii) fasting serum glucose ≥ 100 mg/dL or diagnosis of T2DM or receiving treatment for T2DM; (iii) blood pressure ≥ 135/85 mmHg or taking specific antihypertensive medication; (iv) plasma triglycerides ≥ 150 mg/dL or undergoing lipid-lowering therapy; (v) plasma high-density lipoproteins (HDL) ≤ 40 mg/dL or undergoing lipid-lowering therapy [26]. The patient met the criteria outlined in Table 2 and was therefore diagnosed with MASLD.

The subject was treated with ursodeoxycholic acid 450 mg 1/daily and a low-fat, high-fiber diet following the Mediterranean dietary regimen for 6 months, in accordance with international guidelines for MASLD treatment [26,27,28,29,30]. This therapeutic approach resulted in a reduction in liver steatosis from moderate (Figure 3) to mild after 6 months, which was confirmed with ultrasound (no ultrasound image available). Currently, the patient is stable from a hepatic and metabolic standpoint and is continuing follow-up at our Centers.

**Figure 4:**
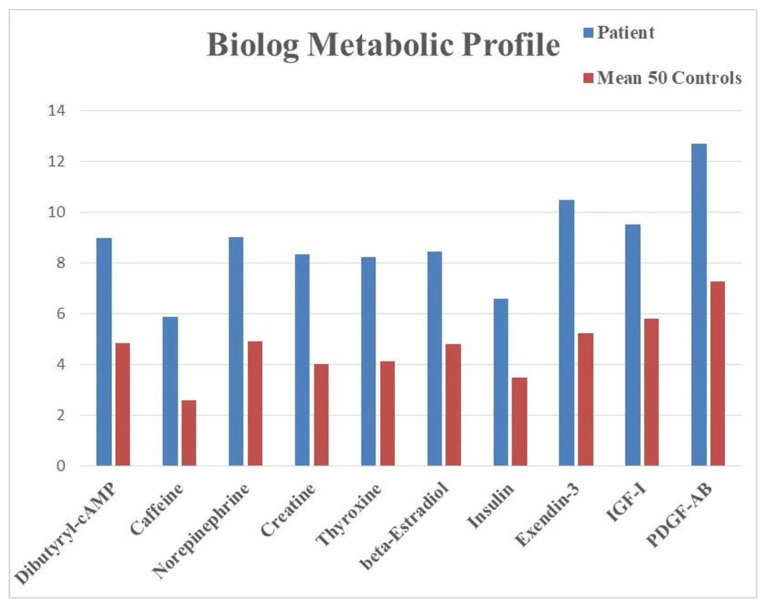
Metabolic profile obtained from LCLs tested on Biolog PM-Ms. The graphic shows endpoint absorbance values (*y* axis) indicating the levels of NADH produced from glucose in the presence of the listed compounds (*x* axis). All differences had an unadjusted *p*-value < 0.1. Abbreviations: IGF-1, insulin-like growth factor-1; PDGF-AB, platelet-derived growth factor-AB.

Metabolic profiling of LCLs from the patient measured the production of energy in the form of NADH in the presence of various compounds, assessing the capacity of the cells to generate energy in response to different metabolic conditions. When compared to the profile of 50 controls, the patient cells showed increased energy production when exposed to several compounds (Figure 4). Even if no difference reached statistical significance (*p*-value < 0.05) due to the comparison of a single case sample versus a control cohort, the results indicated insightful trends with unadjusted *p*-values < 0.1 and suggested possible alterations of multiple metabolic pathways, ranging from hormones (thyroxine or T4, beta-estradiol, insulin) to growth factors (insulin-like growth factor-1, IGF-1, platelet-derived growth factor-AB, PDGF-AB).

The data emerging from the metabolic profile indicate an increased responsivity of the patient’s cells to hormones and growth factors like thyroxine (T4), insulin, IGF-1, and PDGF-AB that promote cellular growth, proliferation, protein synthesis, and storage of energy sources. These metabolic findings indicate that LCLs from the proband tend to increase their production of NADH more than control cells when exposed to hormones or growth factors promoting tissue building and fat accumulation. This trend suggests a predisposition towards an anabolic profile and is in line with the observed clinical presentation, characterized by obesity and liver steatosis. Recognizing a genetic predisposition to an anabolic profile bears relevant translational implications for the clinical and dietary management of the patient.

Another relevant trend is characterized by the increased response to stimulatory compounds, such as dibutyril-cAMP, caffeine, norepinephrine, and creatine. The elevated energy production in LCLs in the presence of such compounds may be compatible with an impaired metabolic activity, which would require a disproportionate amount of energy sources.

## 4. Discussion

### 4.1. PMS and MASLD Interplay

The reported case with a diagnosis of PMS due to a small deletion on chromosome 22q13.33 and MASLD is likely associated with a homozygous p.I148M variant in *PNPLA3*. Although the two conditions are etiologically independent, the *PNPLA3* gene maps on chromosome 22q13.31 and is often deleted in cases of PMS caused by large chromosomal deletions (>5 Mb, see Figure 2) [11]. Despite its deleterious effects on liver function and lipid metabolism, this variant is relatively common in the general population, making it the main genetic factor predisposing to various liver diseases, including MASLD. In cases of PMS, due to larger deletions, one copy of *PNPLA3* may be deleted. The combination of the p.I148M variant on one allele of *PNPLA3* with the deletion of the other allele would amplify the deleterious effects of the variant. It has been shown that the liver phenotype associated with a combination of a 22q13.3 deletion including *PNPLA3* and a p.I148M variant is virtually indistinguishable from the one caused by a homozygous p.I148M variant [5]. The study presents clinical, imaging, and laboratory data on an individual previously described for his liver phenotype, highlighting the multisystemic evolution of his condition, now satisfying the clinical criteria for MASLD [5]. Functional in vitro studies on cells from the reported case subject revealed abnormal metabolic trends with altered energetic responses to hormones either produced by the liver (like IGF-1) or targeting it (thyroxine). Even if the results failed to reach statistical significance because only one case sample was available, it is plausible to postulate that further studies may validate a potential metabolomic profile characterizing individuals with PMS and homozygous *PNPLA3* variants. The results emerging from this longitudinal assessment underline the complexity of the phenotype associated with *PNPLA3* variants and suggest a pivotal role for this gene in the gastrointestinal phenotype of PMS as well as in the genetic predisposition to liver disease in general and MASLD in particular. The data emerging here reported are in line with the current literature and validate the pivotal role of *PNPLA3* variants in MASLD, highlighting the multisystemic implications of the metabolic disruption caused by the abnormal *PNPLA3* function in hepatocytes [5,16,17,18,19]. In fact, our longitudinal investigation allowed us to describe how the evolution of the *PNPLA3*-associated phenotype incorporates the involvement of signs and symptoms beyond the liver, such as obesity, while other parameters might be within normal ranges thanks to the therapeutic regimen. These findings indicate that individuals with PMS caused by large (>5 Mb) 22q13.3 deletions may be at risk for liver disease and, in the long term, MASLD. The same predisposition may affect individuals with homozygous *PNPLA3* variants only. The dynamic phenotype associated with variants in this gene also emphasizes the need for a multidisciplinary approach to MASLD, with guidelines focusing on monitoring lipid and glucose metabolism and the cardiovascular system. Moreover, in cases of large 22q13.3 deletions where *PNPLA3* may be deleted, clinicians should investigate whether the deletion also encompasses the *CYP2D6* gene, one of the most critical pharmacogenes, mapping on chromosome 22q13.2 (Figure 2) [8,11,31]. Loss of one active copy of *CYP2D6*, in the presence of impaired liver function, may increase the risk for adverse reactions to medications, complicating the overall phenotype [32,33].

### 4.2. Strengths and Limitations

Our report presents several notable strengths. First, it integrates detailed clinical, genetic, imaging, and metabolomic data from a single patient with PMS carrying a homozygous *PNPLA3* p.I148M variant, thereby providing a unique longitudinal perspective. The availability of repeated clinical assessments over time, including neurodevelopmental, metabolic, and hepatological parameters, allows for a dynamic evaluation of the patient’s phenotype. Such a longitudinal approach is rarely available in rare genetic syndromes, and it offers valuable insights into the natural history of disease progression and therapeutic response. Second, the use of LCLs and PM-Ms technology enabled functional testing beyond descriptive clinical observation. This methodological strength provided preliminary evidence of altered metabolic responses to hormones and growth factors, which may represent a candidate biomarker signature for patients with PMS and *PNPLA3* variants. Although the presented results are promising, they were generated on a single patient and require validation on a much larger cohort. Third, the incorporation of imaging data and compliance with internationally accepted MASLD diagnostic criteria strengthen the reliability of the liver disease diagnosis and its correlation with genetic findings. Finally, the present report contributes to a growing body of literature emphasizing the multisystemic impact of *PNPLA3* variants, underlining the importance of considering liver disease surveillance in syndromic patients. Nevertheless, important limitations must be acknowledged. Foremost, this is a single-case study, which inherently limits generalizability: we aimed to perform an exploratory longitudinal study to monitor the possible evolution of the gastrointestinal and metabolic phenotype in an individual with PMS. Although the genetic predisposition to abnormal liver function reported in this condition makes it a good model disorder for MASLD and other liver diseases, the rarity and phenotypical variability of PMS suggest that further validation is necessary before translating these results to clinical protocols. Indeed, observed associations between PMS, *PNPLA3* homozygosity, and MASLD must therefore be interpreted with caution until confirmed in larger cohorts. The absence of a family-based genetic analysis also prevents a more complete understanding of inheritance patterns and penetrance in this case. Furthermore, the metabolic profiling findings, while suggestive, did not achieve statistical significance after correction for multiple testing. This limitation reflects both the low statistical power of a single sample and the technical variability intrinsic to in vitro assays. Another limitation is the role of ultrasound imaging as the primary tool in the follow-up of liver steatosis. Although widely available and noninvasive, ultrasound lacks the sensitivity of advanced imaging modalities such as transient elastography, which could have provided more accurate quantification of hepatic fat and fibrosis. Finally, environmental and lifestyle factors, including adherence to the Mediterranean diet, may have influenced the clinical course but could not be rigorously quantified in our investigation.

## 5. Conclusions and Future Perspectives

Although PMS is a relatively rare genetic disorder, the clinical and functional investigation of the phenotypes associated with mutations of the genes in the 22q13.3 region may bring to light valuable genotype–phenotype correlations that may apply to non-syndromic cases as well. In our experience, we provided further evidence supporting the pathogenic role of *PNPLA3* variants in the pathogenesis of MASLD in a subject with PMS, suggesting that the phenotype would evolve with time and highlighting the importance of a thorough multidisciplinary surveillance protocol in individuals with large 22q13.3 deletions. Building on our findings, several valuable avenues for future research emerge. First, expanding the study to larger cohorts of individuals with PMS, especially those harboring *PNPLA3* variants, would help establish genotype–phenotype correlations and assess the generalizability of our observations in MASLD evolution. Indeed, large-scale epidemiological and sequencing studies have demonstrated that the *PNPLA3* I148M variant is the most impactful genetic determinant of MASLD worldwide, responsible for significant variation in disease severity and outcomes across populations [34]. Second, exploring the mechanistic underpinnings of *PNPLA3*-driven hepatic pathology in PMS is critical. A recent study has revealed an association between the *PNPLA3* I148M variant and increased immune cell infiltration and activation in MASLD onset, highlighting a possible inflammatory axis to disease progression [19]. Delineating these immune-mediated pathways could unveil novel therapeutic targets. Third, the advent of precision-medicine approaches, such as RNA silencing therapies targeting *PNPLA3*, offers promising translational potential. Preclinical models and early-phase trials have already demonstrated a reduction in liver fat content in carriers of the risk allele, supporting the feasibility of this intervention [35]. At the same time, ARO-PNPLA3, an RNA interference–based therapy, was recently designed to reduce hepatic *PNPLA3* expression in patients with MASH. Specifically, in a Phase 1 clinical trial (NCT04844450), a single-dose treatment produced encouraging results, achieving dose-dependent reductions of nearly 40% in liver fat content among individuals homozygous for the I148M mutation [36]. Finally, integrating polygenic risk scores that include *PNPLA3* alongside clinical scores (e.g., Fibrosis-4 index) could refine risk stratification for liver-related events in MASLD. In longitudinal cohorts, combined genetic-clinical models have shown markedly improved prediction of adverse outcomes [37]. In addition, future research should also investigate environmental and lifestyle modifiers that could interact with *PNPLA3*-driven susceptibility in PMS. Diet, physical activity, and alcohol consumption are well-established contributors to MASLD severity, and their potential synergistic or mitigating effects in genetically predisposed individuals remain incompletely understood. For example, adherence to a Mediterranean diet has been linked to improved hepatic outcomes in *PNPLA3* carriers, suggesting that nutritional interventions may be particularly beneficial in this subgroup of patients [38]. Similarly, studies of gut microbiota composition in PMS patients could provide insight into whether alterations in the gut-liver axis further exacerbate *PNPLA3*-related hepatic vulnerability [39]. Another important direction lies in longitudinal natural-history studies of PMS individuals carrying *PNPLA3* variants. Such efforts would clarify whether the onset of MASLD-related complications, such as fibrosis progression or HCC, follows a predictable trajectory, and whether early lifestyle or pharmacological interventions can alter this course [40]. Importantly, incorporating advanced imaging modalities (e.g., transient elastography, contrast-enhanced ultrasound, or magnetic resonance imaging-based biomarkers) could improve noninvasive monitoring and reduce reliance on liver biopsy [41]. Ultimately, these integrated approaches underscore the importance of multidisciplinary care in PMS patients, encompassing genetics, hepatology, neurology, and nutrition. By characterizing the complex interplay between genetic and environmental factors, and by translating mechanistic insights into therapeutic strategies, future studies may substantially improve both the quality of life and long-term outcomes of individuals with PMS who are at risk for MASLD [42]. Moreover, the clinical significance of integrating genetic counseling into the management of PMS cannot be overlooked. Families affected by large 22q13.3 deletions would greatly benefit from structured counseling programs that include information on MASLD risk, lifestyle modifications, and potential participation in clinical trials targeting *PNPLA3*. Educational initiatives could also empower caregivers to recognize early metabolic or hepatic warning signs, prompting timely specialist referral [43]. From a healthcare perspective, these measures would not only enhance personalized care but also generate valuable real-world evidence to guide broader recommendations for rare genetic syndromes intersecting with common metabolic diseases. Overall, the results emerging from this study describe the longitudinal evolution of the liver phenotype from steatosis to MASLD in a subject with PMS and provide functional evidence of pathogenic mechanisms based on a predisposing genetic background. Although these findings were reported in a patient with a rare genetic disorder, it is plausible that similar mechanisms and clinical patterns apply to sporadic cases as well, providing relevant information about the risk of MASLD and the possible evolution of the liver phenotype in predisposed subjects.

## Figures and Tables

**Table 1:** Summary of the clinical manifestations in patients with PMS.

Domain	Clinical Manifestations
Neurological/Developmental	Intellectual disability Developmental delay Language delay or absent speech Motor impairment Hypotonia Seizures Sleep disturbances Autism spectrum disorder/autistic traits
Systemic	Gastrointestinal diseases Kidney diseases Lymphedema
Hepatic/Metabolic	MASLD Metabolic syndrome
Other features	Minor dysmorphic traits

Abbreviations: MASLD, metabolic-associated steatotic liver disease.

**Table 2:** MASLD criteria—SLD plus at least one of five cardiometabolic risk factors.

MASLD Criterium	Value	Satisfied
SLD	Moderate SLD	Yes
BMI ≥ 25 kg/m^2^ or waist circumference > 94 cm	BMI 26.57 kg/m^2^ Waist circumference 101 cm	Yes
Fasting serum glucose ≥ 100 mg/dL or diagnosis of T2DM	93 mg/dL	No
Blood pressure ≥ 135/85 mmHg	120/80 mmHg	No
Triglycerides ≥ 150 mg/dL	111 mg/dL	No
HDL ≤ 40 mg/dL	62 mg/dL	No

Abbreviations: SLD: steatotic liver disease; BMI: body mass index; T2DM: type 2 diabetes mellitus; HDL: high-density lipoproteins.

## Data Availability

All data are available in the manuscript.

## Abbreviations

The following abbreviations are used in this manuscript:

BMI	Body mass index
CYP2D6	Cytochrome P450 2D6
HCC	Hepatocellular carcinoma
HDL	High-density lipoproteins
IGF-1	Insulin-like growth factor 1
LCLs	Lymphoblastoid cell lines
MASH	Metabolic dysfunction-associated steatohepatitis
MASLD	Metabolic-associated steatotic liver disease
NAFLD	Non-alcoholic fatty liver disease
NADH	Nicotinamide adenine dinucleotide
PDGF-AB	Platelet-derived growth factor-AB
PMS	Phelan–McDermid Syndrome
PNPLA3	Patatin-like phospholipase domain containing 3
PM-Ms	Phenotype Mammalian Microarrays
SLD	Steatotic liver disease
SNP	Single nucleotide polymorphism
T2DM	Type 2 diabetes mellitus
